# Supplementary figures and images for: AP003352.1/miR-141-3p axis enhances the proliferation of osteosarcoma by LPAR3
Source: PeerJ. 2023 Sep 15;11:e15937. doi: 10.7717/peerj.15937 (PMC10506581; doi:10.7717/peerj.15937)

Figure4E

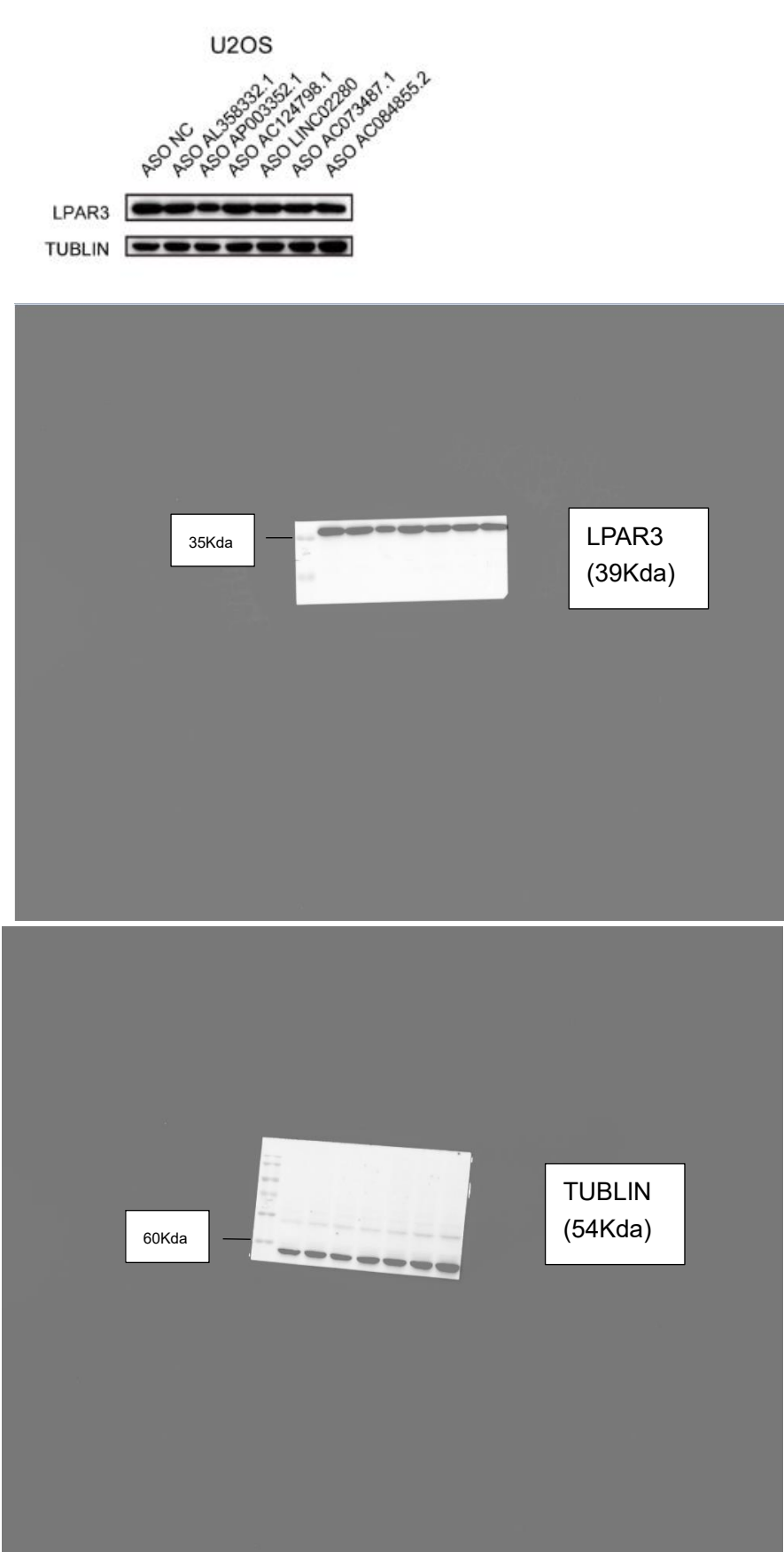

Figure6A

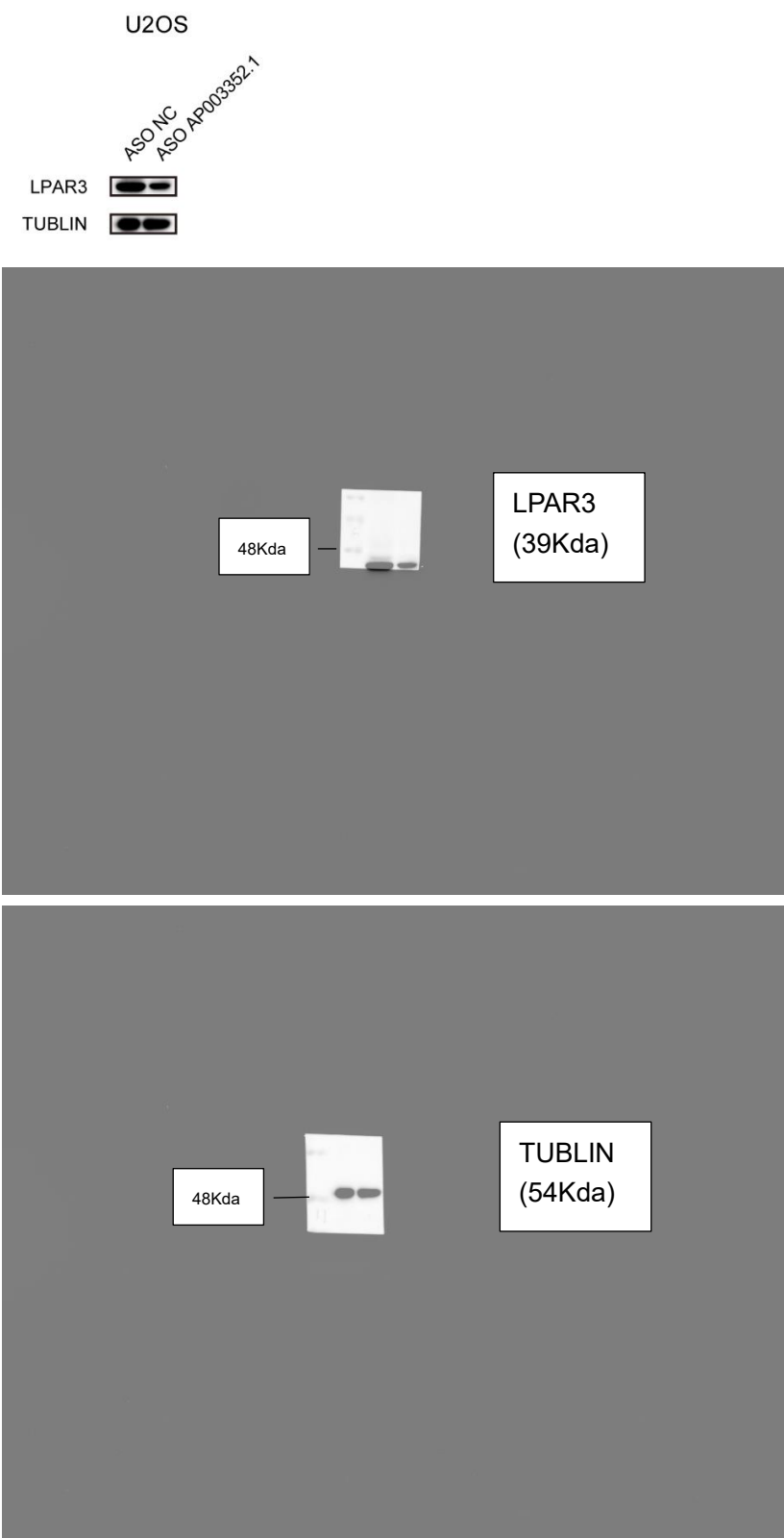

Figure6B

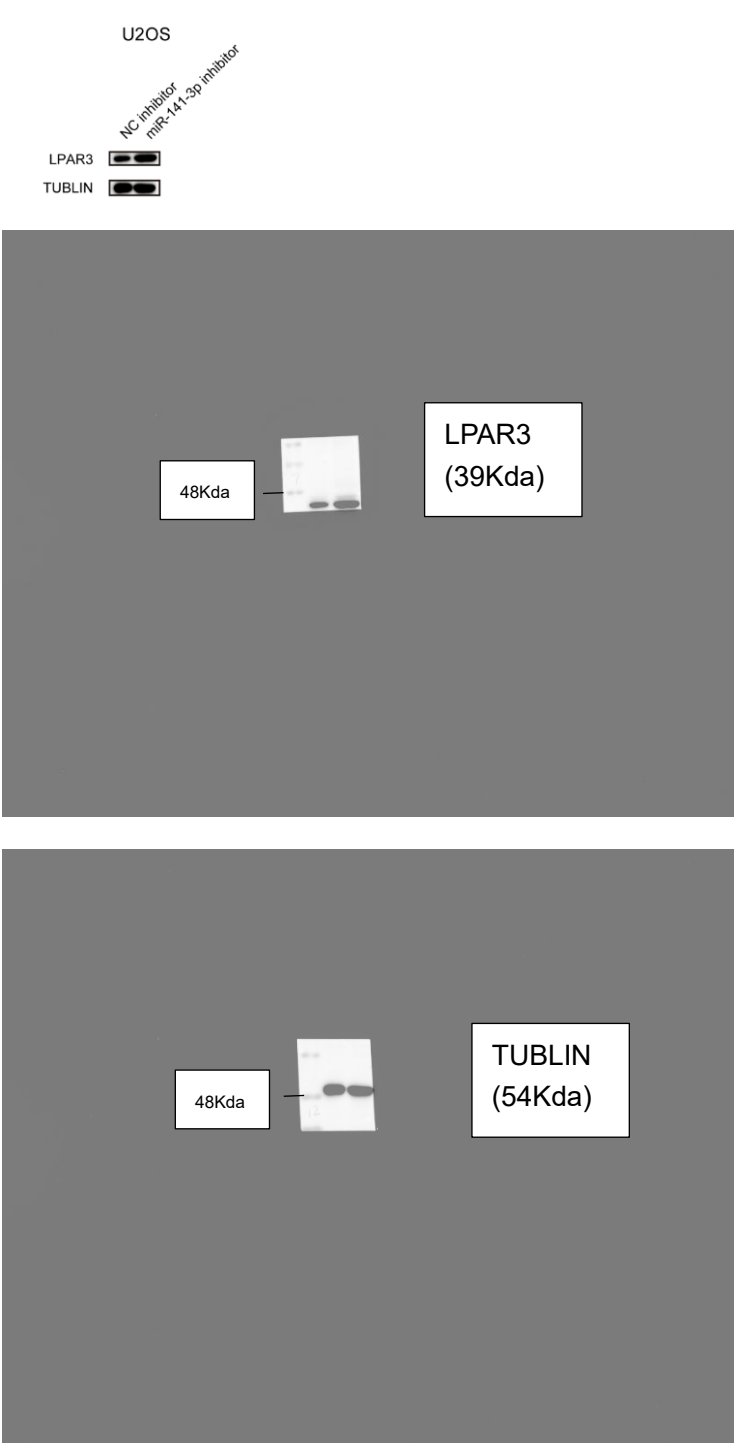

Figure6C

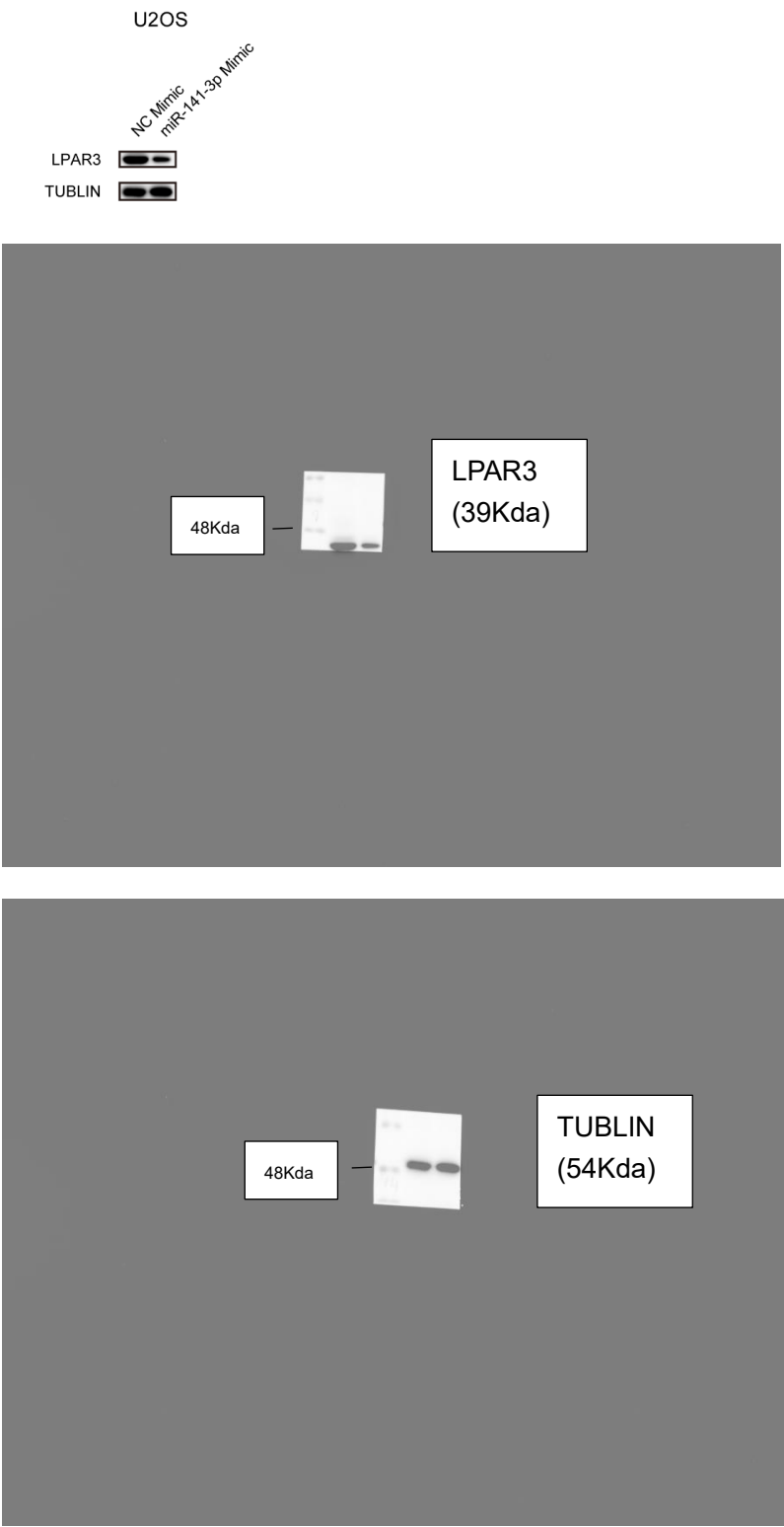

Supplement: Supplemental Information 1 [file peerj-11-15937-s001.pdf]

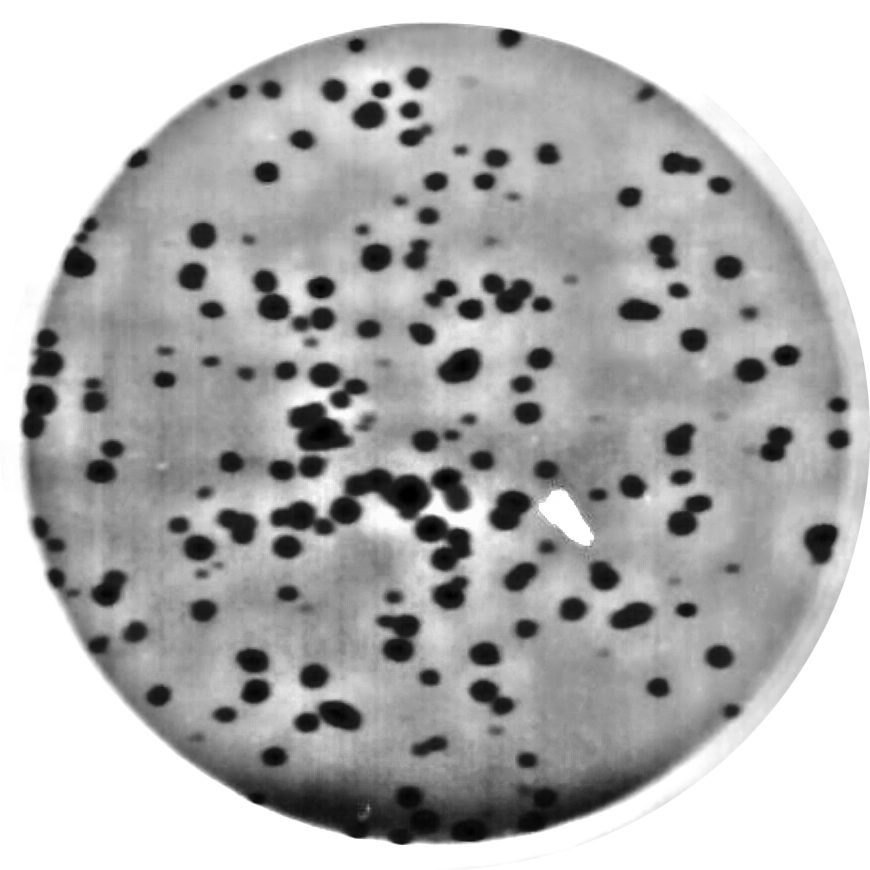

Supplement: Supplemental Information 2 [file peerj-11-15937-s002.zip › Original Figure5/C/lncRNA/ASO-AP003352.1.tif]

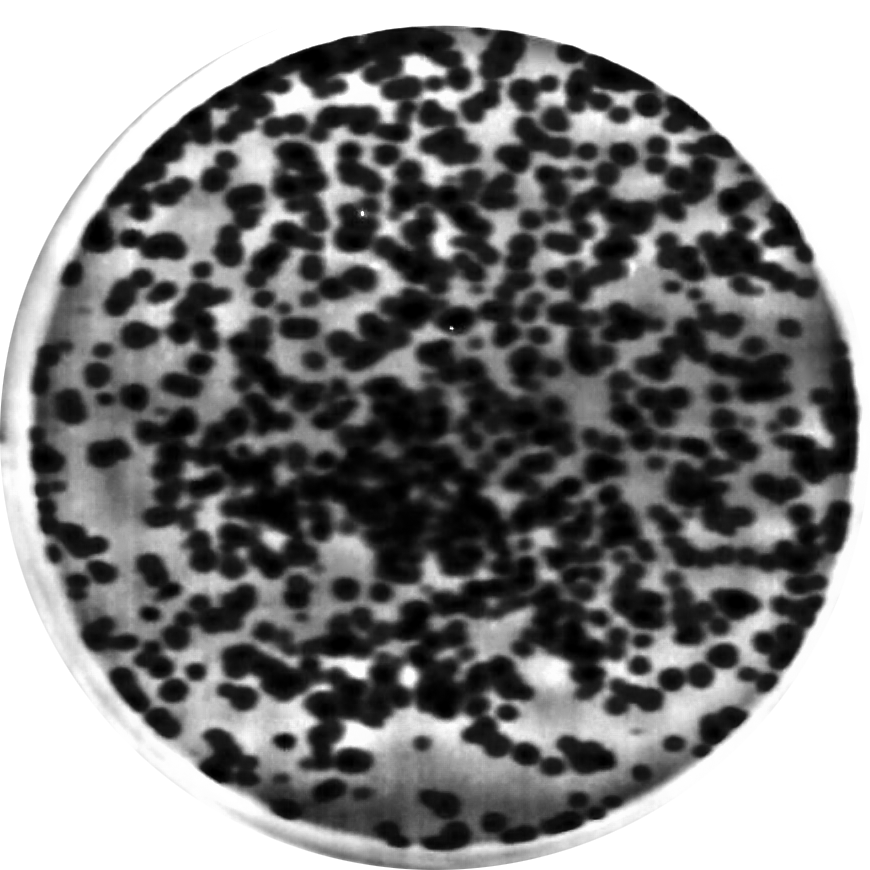

Supplement: Supplemental Information 2 [file peerj-11-15937-s002.zip › Original Figure5/C/lncRNA/ASO-NC.tif]

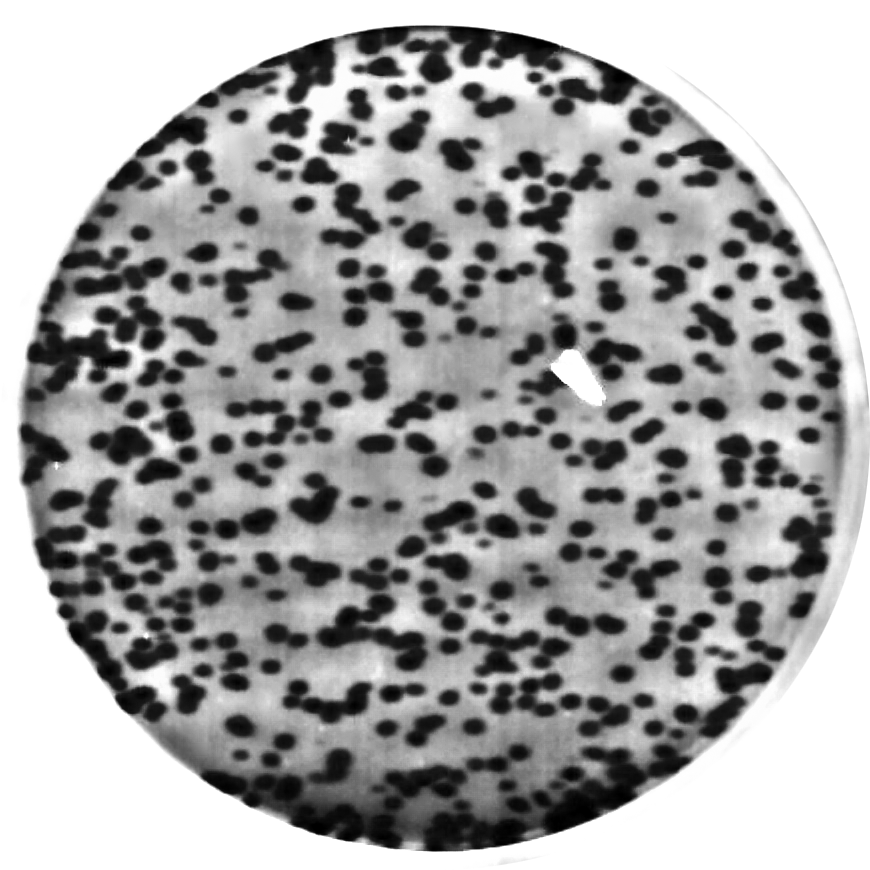

Supplement: Supplemental Information 2 [file peerj-11-15937-s002.zip › Original Figure5/C/microRNA/miRNA-inhibitor.tif]

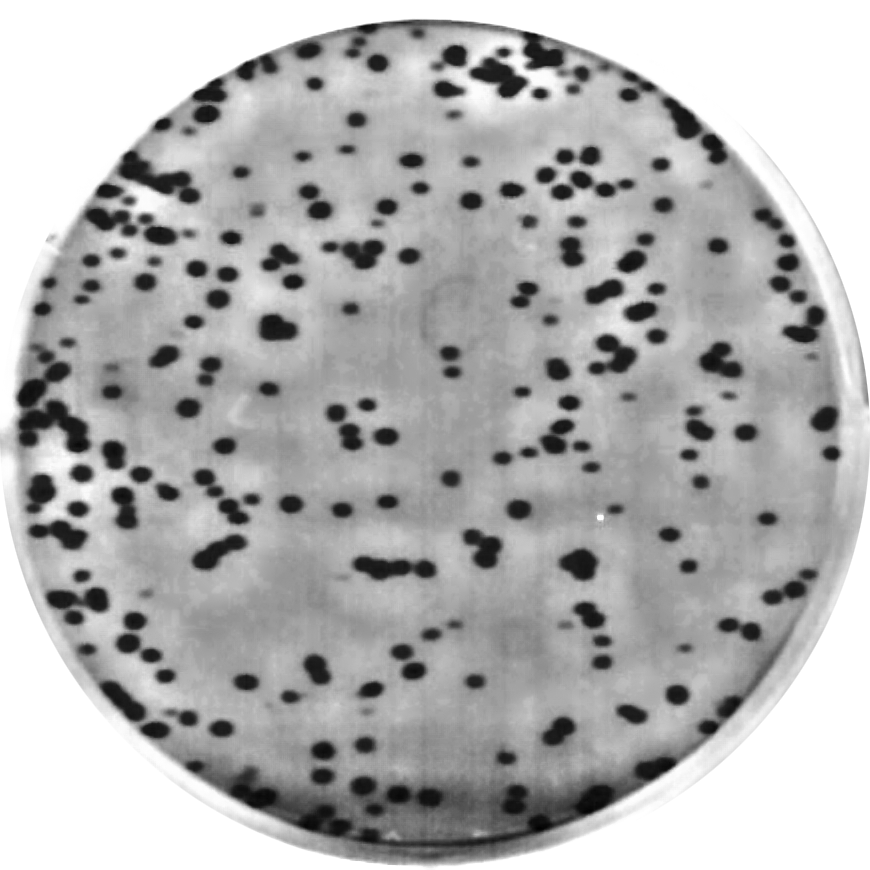

Supplement: Supplemental Information 2 [file peerj-11-15937-s002.zip › Original Figure5/C/microRNA/miRNA-NC.tif]

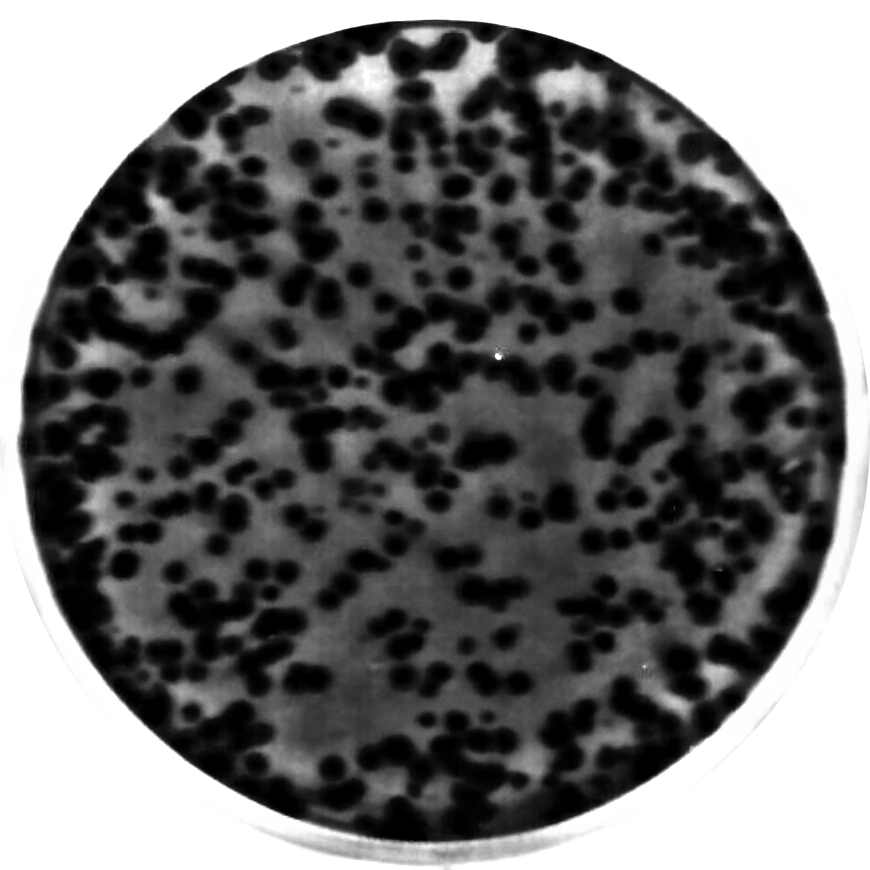

Supplement: Supplemental Information 2 [file peerj-11-15937-s002.zip › Original Figure5/C/mRNA/shLPAR3.tif]

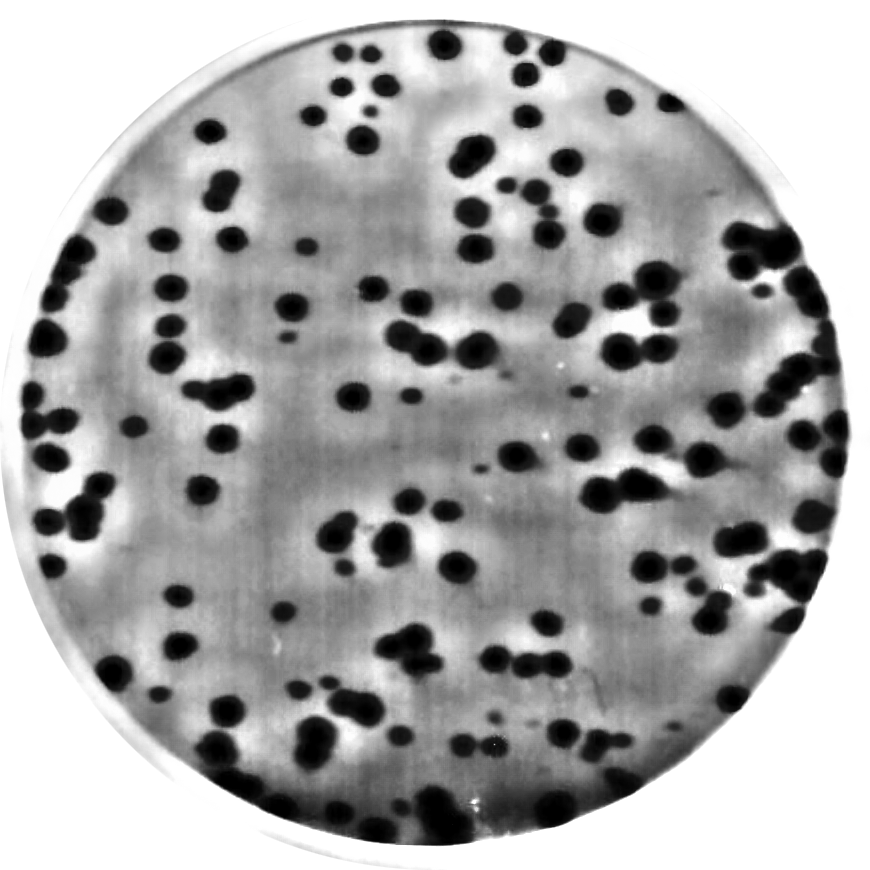

Supplement: Supplemental Information 2 [file peerj-11-15937-s002.zip › Original Figure5/C/mRNA/shNC.tif]

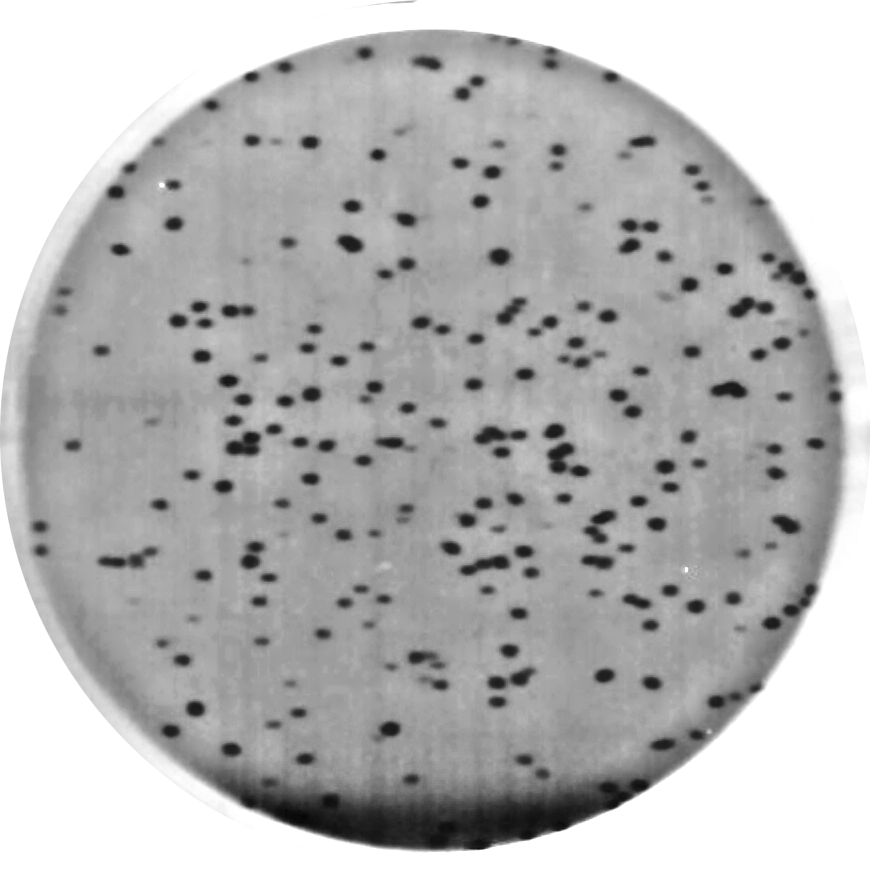

Supplement: Supplemental Information 2 [file peerj-11-15937-s002.zip › Original Figure7/D/rescue/ASO lnc+mimic miR.tif]

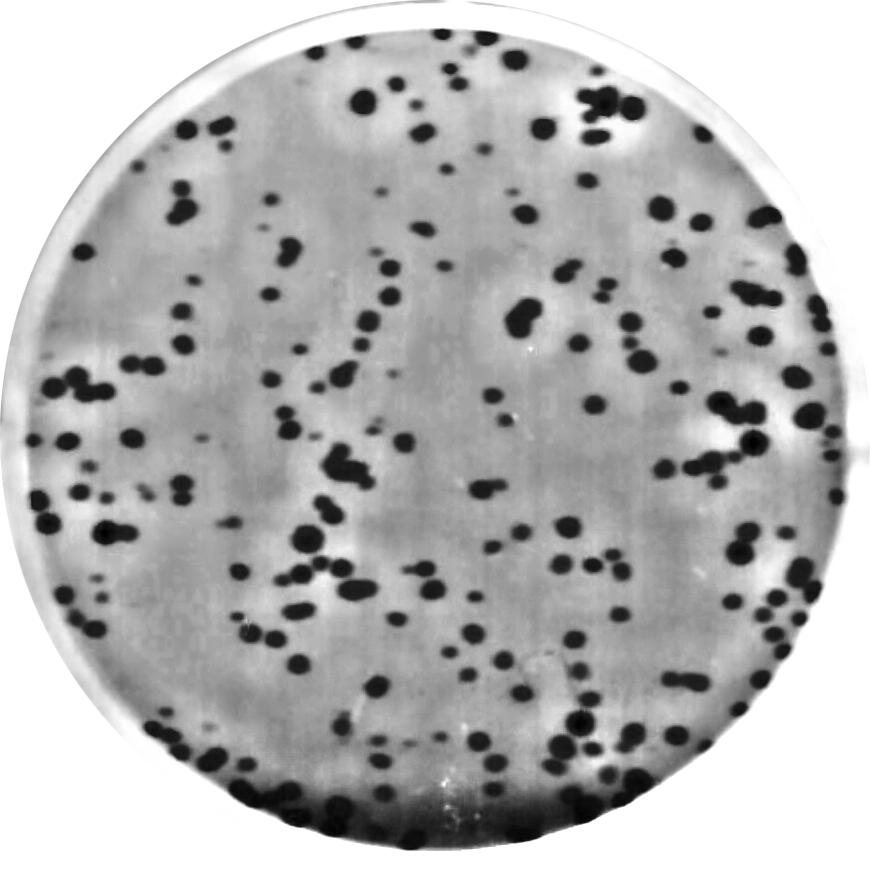

Supplement: Supplemental Information 2 [file peerj-11-15937-s002.zip › Original Figure7/D/rescue/ASO lnc+mimic NC.tif]

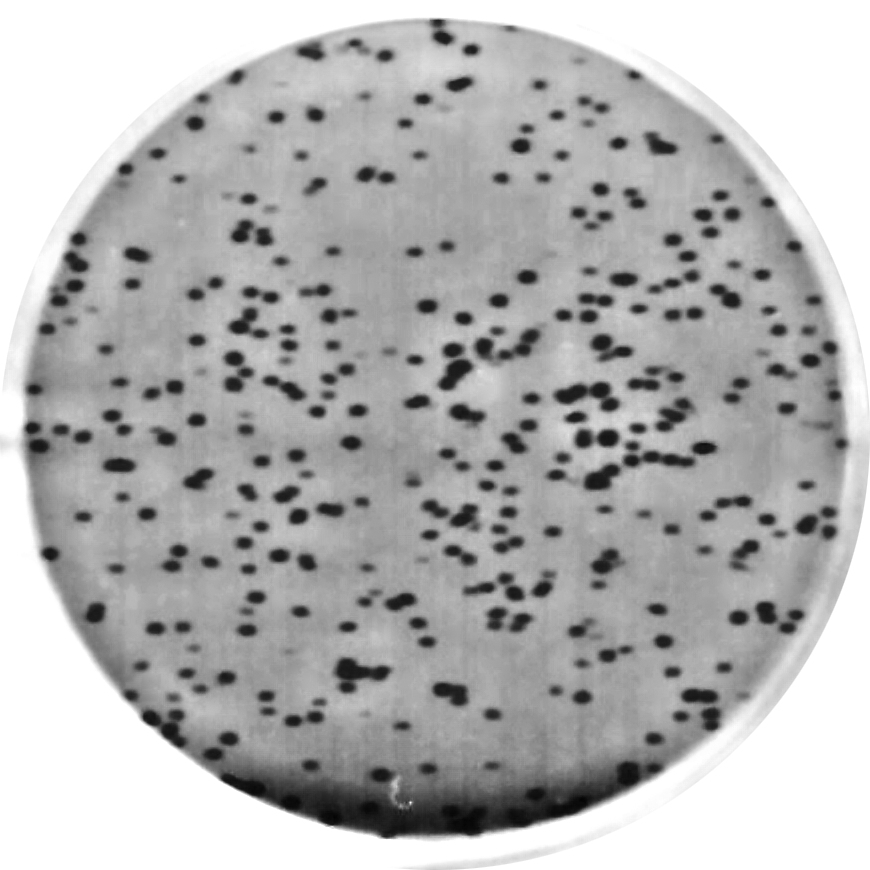

Supplement: Supplemental Information 2 [file peerj-11-15937-s002.zip › Original Figure7/D/rescue/ASO NC+mimic miR.tif]

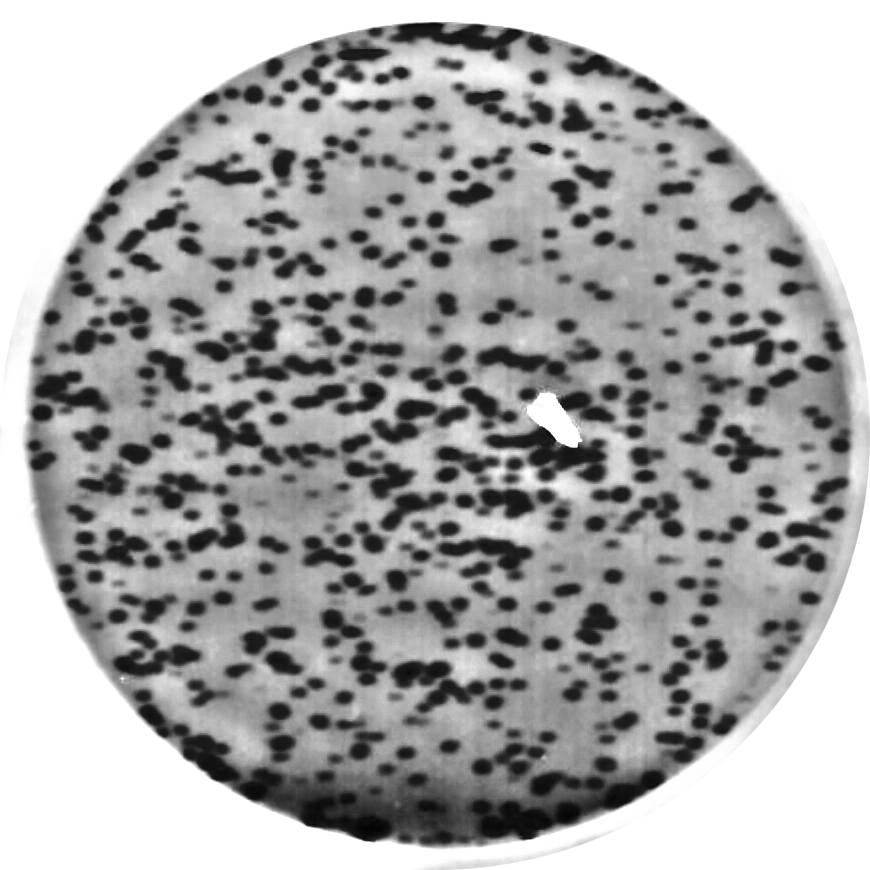

Supplement: Supplemental Information 2 [file peerj-11-15937-s002.zip › Original Figure7/D/rescue/ASO NC+mimic NC.tif]
