# Supplementary material for: AP003352.1/miR-141-3p axis enhances the proliferation of osteosarcoma by LPAR3
Source: PeerJ. 2023 Sep 15;11:e15937. doi: 10.7717/peerj.15937 (PMC10506581; doi:10.7717/peerj.15937)
Supplement: Supplemental Information 3 [file peerj-11-15937-s003.doc]

**FigureS1**

**
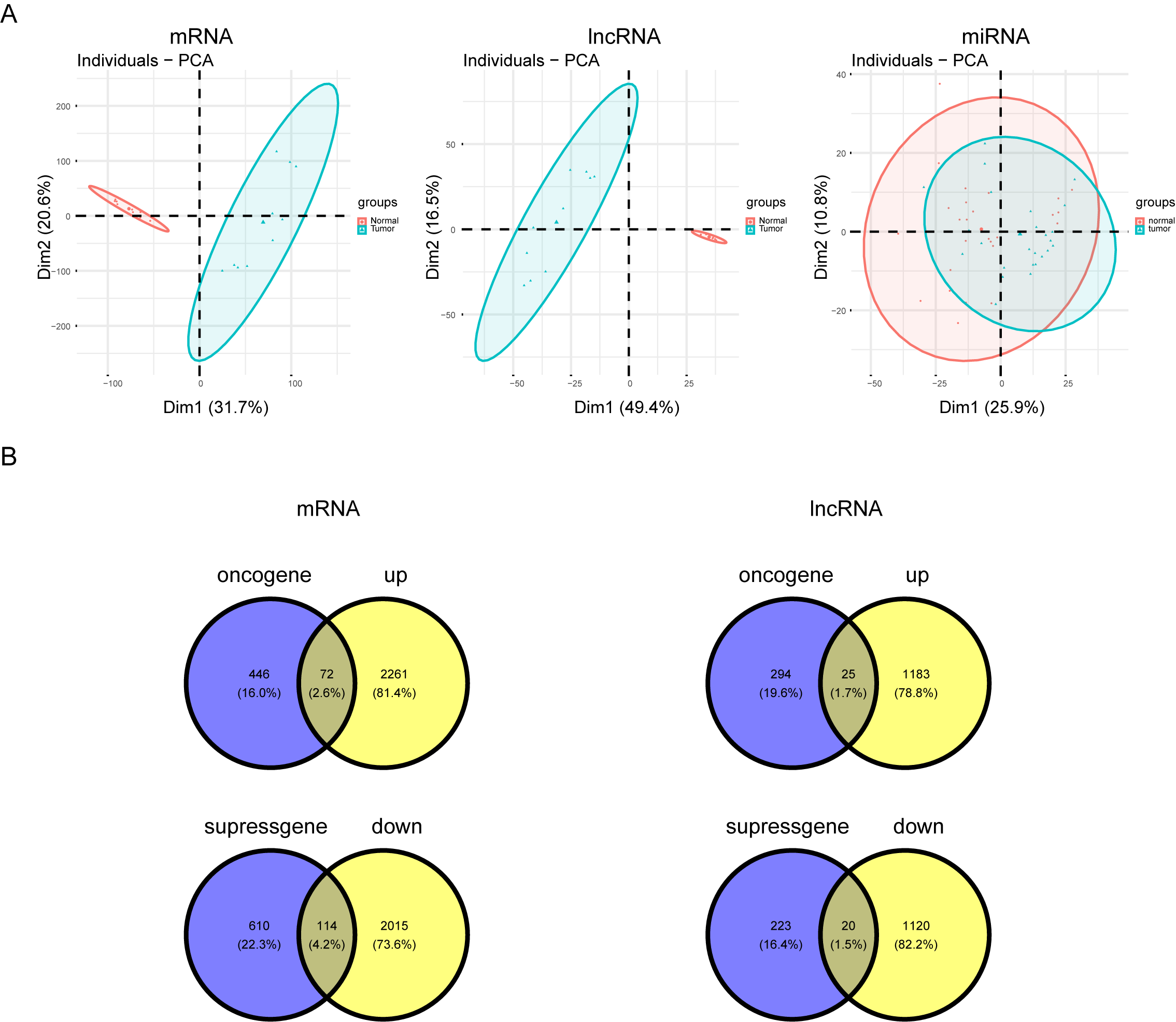
**

**FigureS2**

**
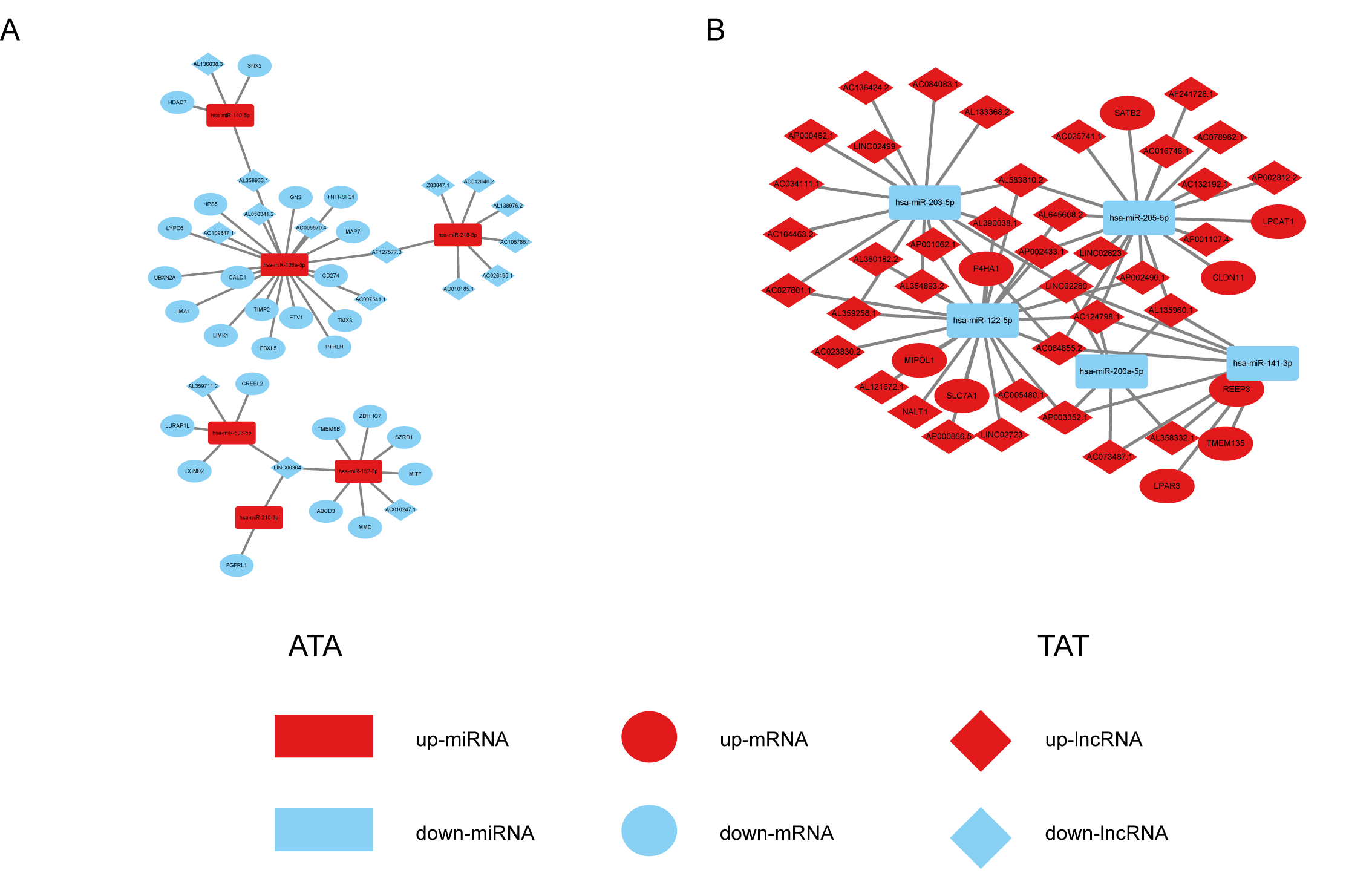
**

**FigureS3**

**
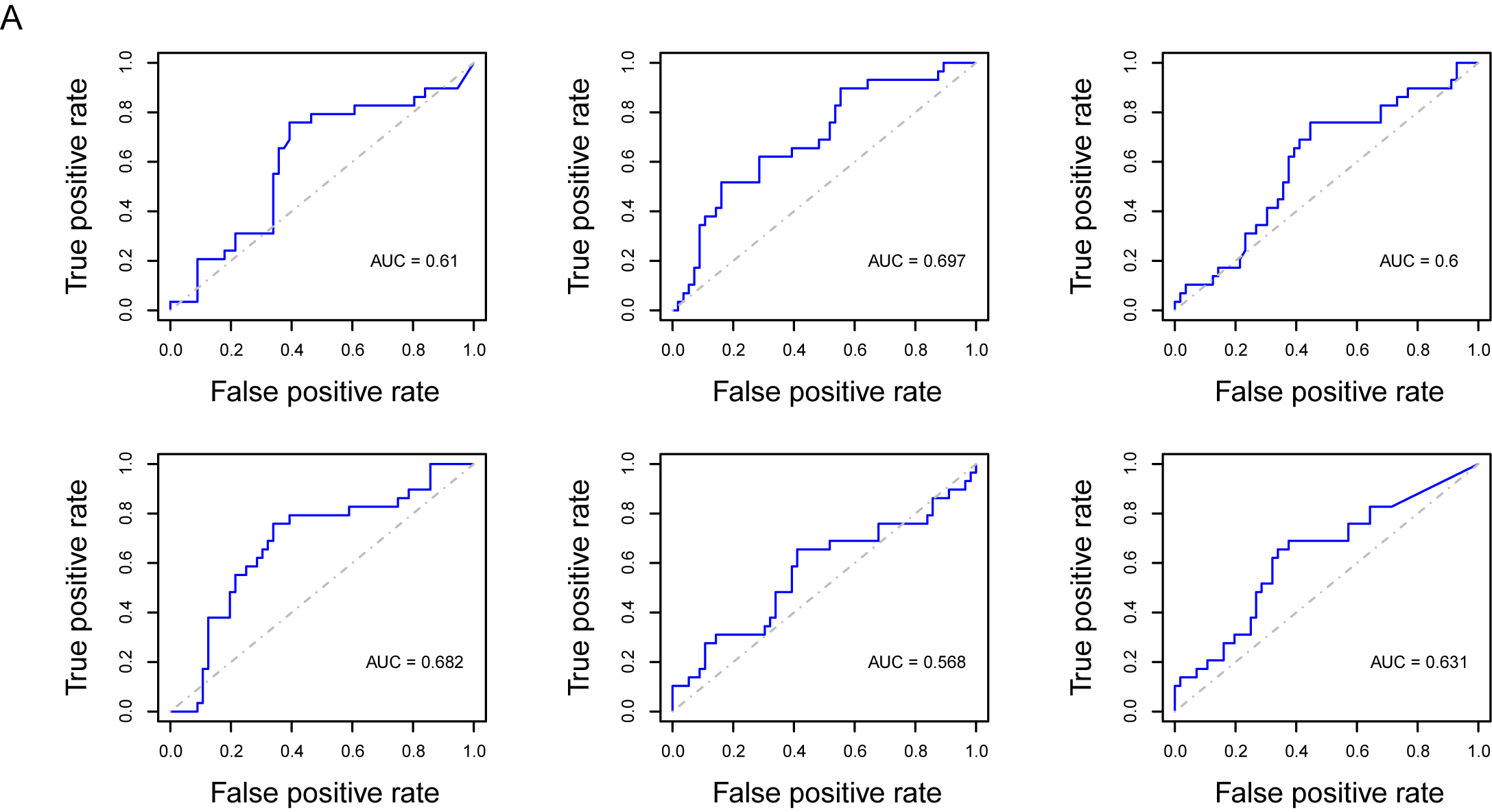
**

**FigureS4**

**
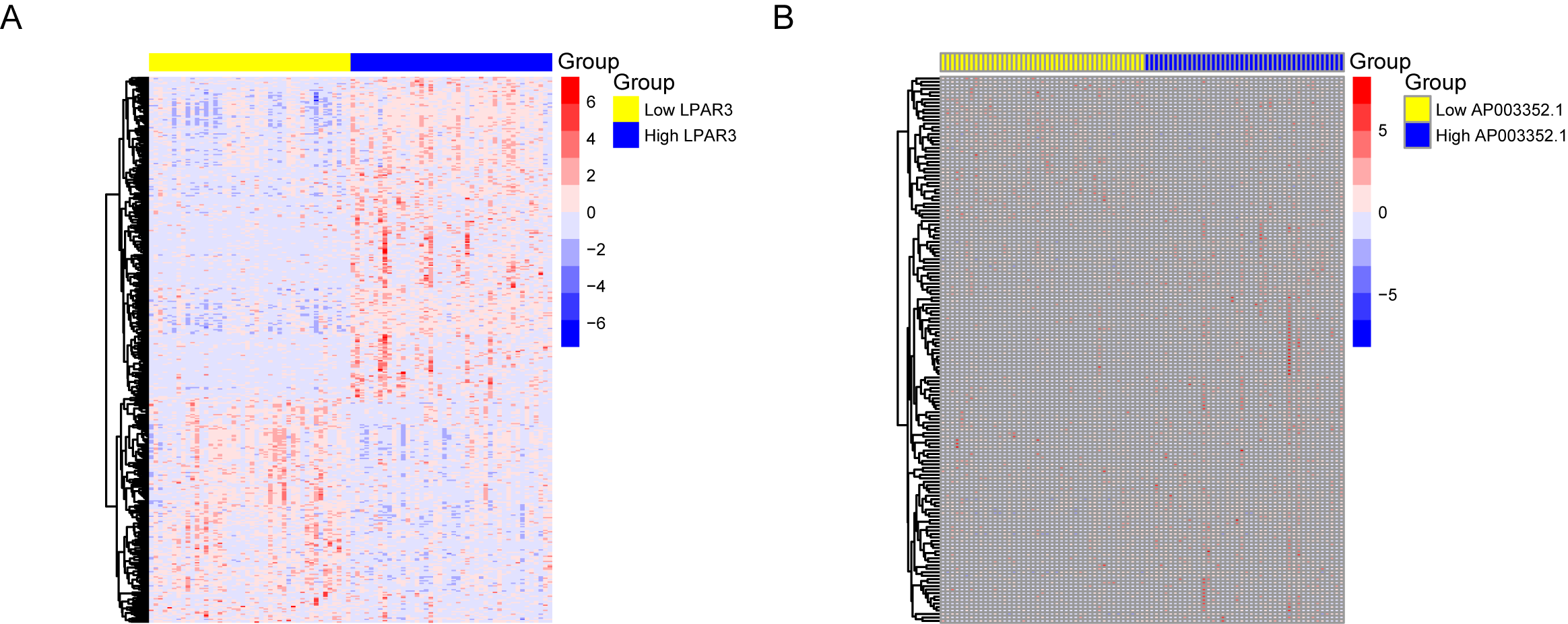
**

**FigureS5**

**
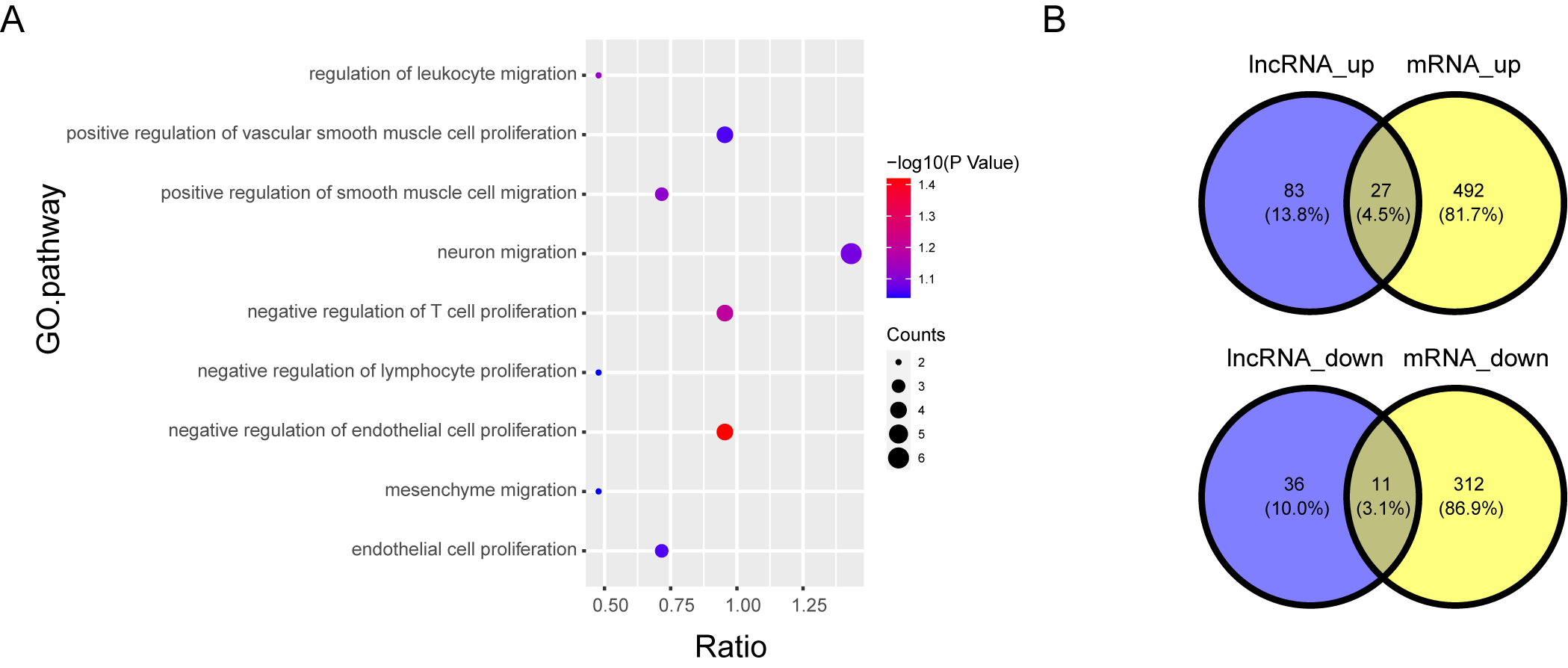
**

**FigureS6**

**
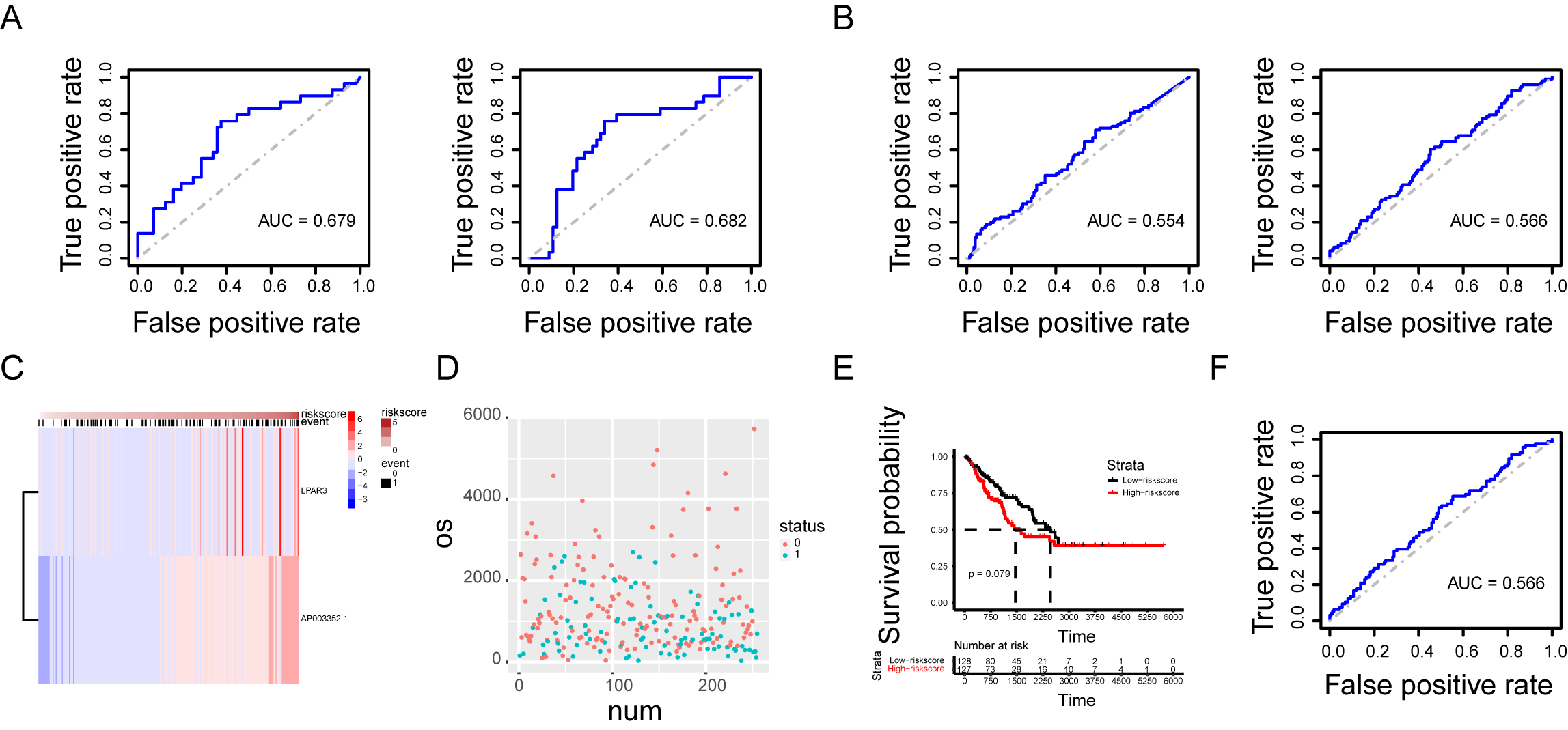
**

**Supplementary figure legends**

**FigureS1**

The results of differential expression analysis of mRNAs, lncRNAs and miRNAs. PCA diagrams of mRNAs, lncRNAs and miRNAs (A). Venn diagrams represent the overlapped genes (B).

**FigureS2**

Construction of ceRNA network by integrated analysis.

**FigureS3**

The ROC curves of the 6 lncRNAs in osteosarcoma (A).

**FigureS4**

The heatmaps of the DEGs in low expression group and high expression group among AP003352.1 and LPAR3.

**FigureS5**

The bubble diagram of GO enrichment analysis of AP003352.1 and LPAR3 overlapped genes by DAVID database (A). Venn diagrams represent the overlapped genes between AP003352.1 and LPAR3 (B).

**FigureS6**

ROC curves of LPAR3 and AP003352.1 in the training cohort (A). ROC curves of LPAR3 and AP003352.1 in the validation cohort (B). The heatmap based on the risk score in the validation cohort (C). Survival status plot of the validation cohort (D). Survival curves for the two groups in the validation cohort (E). ROC curves based on the risk score in the validation cohort (F).
